# Study on Carbonation of Porcine Blood Hydrogel in the Composite Mortar of Ancient Chinese Architectural Painting

**DOI:** 10.3390/gels10030191

**Published:** 2024-03-09

**Authors:** Cong Cheng, Wenhua Ma, Rui Chen, Yeting Zhu, Lizhen Zheng, Wei Li, Daodao Hu

**Affiliations:** 1 Engineering Research Center of Historical Cultural Heritage Conservation, Ministry of Education, School of Materials Science and Engineering, Shaanxi Normal University, Xi’an 710119, China; congcheng2017@snnu.edu.cn (C.C.); mawh@snnu.edu.cn (W.M.); crcherie@snnu.edu.cn (R.C.); zyeting@snnu.edu.cn (Y.Z.); 2 School of Historical Culture and Tourism, Xi’an University, Xi’an 710065, China; lizhenzheng@snnu.edu.cn; 3 Shaanxi Engineering Research Center of Controllable Neutron Source, School of Electronic Information, Xijing University, Xi’an 710123, China

**Keywords:** porcine blood hydrogel, traditional mortar, ancient Chinese architectural painting, carbonation, lime water

## Abstract

In the ancient Chinese recipe for composite mortar used in the construction of ground layers for architectural painting, the mixture of porcine blood and lime water is one of the constituent materials. Herein, according to the traditional recipe, the interaction between porcine blood and lime water was systematically and deeply investigated. The experimental investigation demonstrated that porcine blood mixed with lime water at the ratio found in the recipe can form a hydrogel with a hydrophobic surface. During air-drying, the lime water in porcine blood hydrogel can react with CO2 to form calcium carbonate. The crystal morphology of the formed calcium carbonate depends on the surrounding micro-environment of calcium ions in the porcine blood hydrogel. The formed morphology of calcium carbonate includes small calcite crystallites, small graininess calcite crystals with round features, calcite aggregates with layered ladder-like structures, and amorphous calcium carbonate (ACC). Interestingly, the calcium carbonate formed in the inner part of the porcine blood hydrogel exhibits lamellar distribution due to a Liesegang pattern formation. Based on the findings that the porcine blood hydrogel has surface hydrophobicity and brittleness, it can be predicted that in the preparation process of composite mortar for ancient building color painting base course, porcine blood used in the form of a hydrogel is not only easier to be dispersed in hydrophobic tung oil than in liquid porcine blood but also the affinity between porcine blood gel and tung oil is enhanced. As constituent material dispersed in the composite mortar, the layered distribution of calcium carbonate in the porcine blood hydrogel may presumably be beneficial to reduce the internal stress of the composite mortar material.

## 1. Introduction

Ancient Chinese architectural painting is an important feature of ancient Chinese architecture, with great historical, cultural, and artistic value. Generally, these paintings are commonly used not only to decorate the surfaces of columns, beams, walls, and other structural elements but also to protect wood from corrosion [1,2]. This kind of architectural painting encompasses three distinct components, including the pigment layer, the ground layer, and the wooden elements. The ground layer, used as mortar, typically consists of brick ash, lime water, fiber, blood, and tung oil, making it a typical organic–inorganic composite material. This kind of mortar possesses excellent properties, such as strong adhesion, hydrophobicity, weather resistance, crack prevention, and high compressive strength, endowing ancient buildings with durability and stability [3,4]. In this kind of mortar, there are lime-based organic–inorganic composite binders. Due to the presence of organic matter, the shortcomings regarding the water resistance and strength of lime mortars are effectively improved. The many advantages of organic–inorganic composite mortars have led to their widespread use in various applications, including indoor murals [5,6], tomb chamber murals [7], and wooden ships [8].

The earliest organic–inorganic composite building mortar was discovered in China and dates to 2300 BCE [9]. It has been shown that ancient traditional lime-based binders contained organic additives such as glutinous rice [10,11,12,13], tung oil [7,14,15], blood [16,17,18,19], egg white [20,21], sugar [20], gelatin [6,9], and peach gum [2]. Similarly, these lime-based mortars also emerged in other countries around the world. The Cretans added animal glue, eggs, and casein to their decorative mortars to improve the mechanical strength of their architectural structures [22]. Indian mortars included traditional local organic materials to enhance strength and resistance against external environmental forces [23]. In Myanmar, glutinous rice and proteins were added to building mortars to meet higher living requirements [6]. From reports in the literature such as glutinous rice–lime mortar [11,24,25,26,27], tung oil–lime mortar [27,28,29], blood–lime mortar [17,18,19], egg white–lime mortar [20,21,28], and sugar–lime mortar [20,28], the same conclusion that organic–inorganic composite mortars have strong adhesion properties could be drawn. In these natural organic matters, blood–lime mortar has received special attention. A study by Lin et al. [30] found that adhesives prepared from blood treated with alkalis have a bonding strength comparable to that of phenol-formaldehyde under dry conditions. Fang et al. [18] conducted comparative studies between ordinary lime mortar and traditional calcium hydroxide–blood glue mortar. The reaction between blood proteins and calcium hydroxide resulted in a denser mortar structure with reduced cracking. After curing, proteins accumulated on the surface, forming a smooth and dense protective layer that enhanced the material’s waterproofing and weather resistance. Zhang et al. [19] investigated the impact of ox blood on lime-based mortars through TG and ATR. In the early stages of curing, ox blood promoted the formation of amorphous calcium carbonate, which, over time, could transform into vaterite and then calcite.

There have been many studies on animal blood mortar; the mortars in most of these studies are made by mixing animal blood and lime in a nearly equal proportion, which can be used as a binder for building components. However, the ratio and function of the mixture of porcine blood and lime studied in this article are different from the abovementioned porcine blood–lime mortar. According to the traditional recipe for the preparation of ancient Chinese architectural paintings, the hydrogel formed by mixing porcine blood and lime water in a much lower weight ratio of Ca(OH)_2_ to the blood is used as an auxiliary material in preparing coating composite materials, and the formed hydrogel needs to be mixed with tung oil to enhance the performance in the adhesion of tung oil adhesive, improving the convenience of construction operations [31]. In the reported literature, the studies on porcine blood hydrogel mainly focused on composition analysis in ancient Chinese architectural paintings. However, there are few investigations on the mechanism of interaction between porcine blood and lime during the formation of hydrogel. In this study, the following main findings are reported: (1) Porcine blood can be gelled by lime water, and the formed hydrogels may be beneficial for the dispersion of porcine blood into hydrophobic tung oil in the preparation of composite mortar. (2) The surface of the dried hydrogels presents hydrophobicity, and this property may be beneficial to the affinity between porcine blood and hydrophobic tung oil. (3) Lime water in the hydrogels could become calcium carbonate, and this calcium carbonate not only enhances the mechanical properties of the composite mortar but also exhibits in the form of the lamellar distribution in the porcine blood hydrogel. These findings are particularly important for profoundly understanding the role of porcine blood in conventional composites.

## 2. Results and Discussion

### 2.1. The Factors Related to the Formation of Porcine Blood Hydrogel

**Figure 1:**
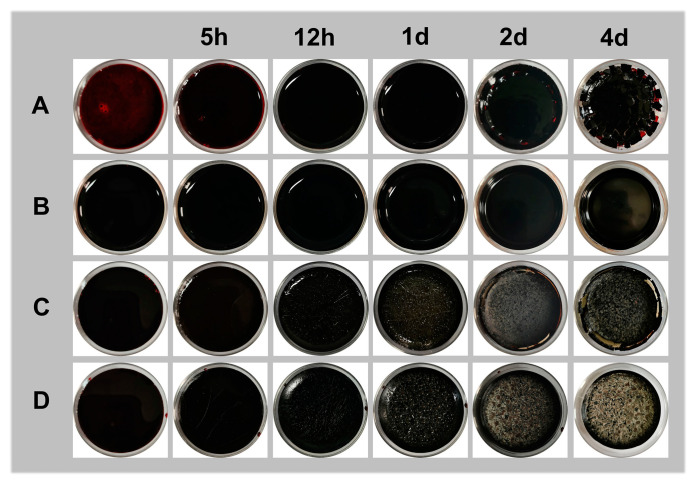
Photos of porcine blood mixed with different additives after standing at the given condition (25–28 °C, 40–60% RH) for different times: (**A**) CaCl_2_, (**B**) NaOH, (**C**) mixture of CaCl_2_ and NaOH, and (**D**) lime water.

Figure 1 illustrates the photos of samples formed by porcine blood mixed with different additives—CaCl_2_ (A), NaOH (C), a mixture of CaCl_2_ and NaOH (C), and lime water (D)—after standing for different times. The detailed compositions are shown in Table 1. At the start, sample A did not form a hydrogel, whereas samples B, C, and D all formed hydrogels. After standing for 4 days, sample A had broken into small fragments; the surface of sample B remained smooth, without any obvious change in morphology; and some white substances appeared in samples C and D. The above results indicate that only calcium ions fail to gelatinize porcine blood. Although both strong alkalis and the coexistence of strong alkalis and calcium ions can cause porcine blood to gel, the final states of their gelation products are significantly different. In fact, the porcine blood hydrogel produced using strong alkalis is very soft due to originating from electrostatic interactions [32]; however, calcium ions can significantly enhance the strength of the porcine blood hydrogels made using strong alkalis. This is related to the cross-linking structure formed by the coordination of calcium ions with carboxyl groups on the porcine blood protein chains [33].

**Figure 2:**
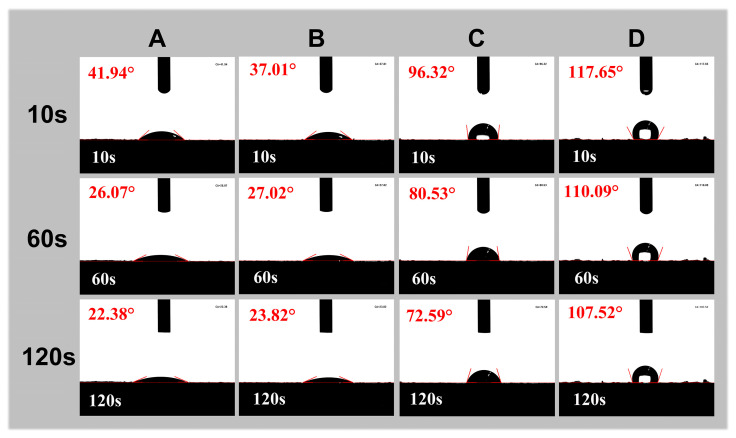
Contact angle images for water droplets on the surfaces of various samples. The powders from the products formed by the mixtures of porcine blood and different additives: (**A**) CaCl_2_, (**B**) NaOH, (**C**) mixture of CaCl_2_ and NaOH, and (**D**) lime water.

Figure 2 shows the contact angles of powders from the products formed by the mixtures of porcine blood and different additives. The products here are the same as the corresponding products mentioned in Figure 1. Obviously, sample D has the maximum contact angle, followed by sample C, while samples A and B have the smallest contact angles. The above results may be related to the conformational changes in porcine blood proteins under the coexistence of calcium ions and alkalis. Under alkaline conditions, deprotonation of carboxyl groups in proteins facilitates coordination with calcium ions, thereby promoting the formation of cross-linked structures. The formation of this cross-linking structure not only enhances the strength of the gel but also exposes the hydrophobic protein chains. As a result, the gel has a strong hydrophobic surface [34].

**Figure 3:**
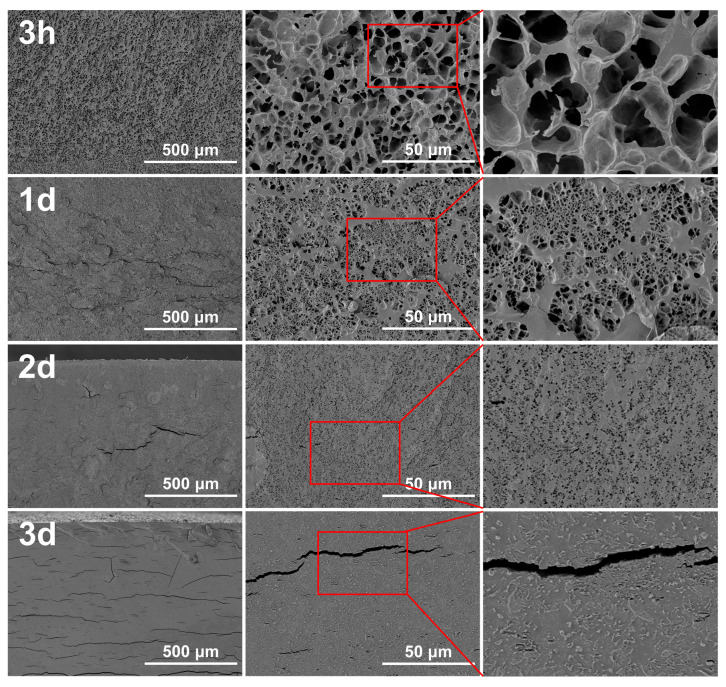
Cross-sectional SEM images (×100, ×1K) of the porcine blood hydrogel after freeze-drying at different times.

Hydrogels are regarded as three-dimensional networks of polymers with the ability to absorb large amounts of water [35]. The material formed by the mixture of porcine blood and lime water is also a hydrogel due to it having hydrogel characteristics. Figure 3 shows the cross-sectional SEM image of the porcine blood hydrogel after freeze-drying at different times. Obviously, the pore structure characteristics of the porcine blood hydrogel disappear as the standing time increases. The freshly prepared hydrogel exhibited a three-dimensional network structure with large pores. After standing for 1 day, 2 days, and 3 days, the pores gradually became denser and smaller until they disappeared.

Inevitably, with the increased times standing in plain air, both the evaporation of water and the carbonation of the lime water promoted the disappearance of the internal pore structure because the carbonation of lime water can fill pores. Porcine blood contains fibrin proteins, and there are rich acidic amino acid residues such as glutamic and aspartic acids on fibrin chains [36,37]. These carboxyl groups can form coordination bonds with calcium ions [38,39,40,41]. As a result, the porcine blood could be gelled by lime water to form the hydrogel. In the formed hydrogel, the distribution of a large amount of water in the cross-linked networks makes the hydrogel porous. Since the prepared porcine blood hydrogel contains Ca(OH)_2_, Ca(OH)_2_ inevitably reacts with CO_2_ in the air to form calcium carbonate when the porcine blood hydrogel is exposed to the air [42]. The changes in morphology characteristics that occurred in the porcine blood hydrogel with time and carbonation are discussed in detail in the next section.

### 2.2. Surface Carbonation on the Porcine Blood Hydrogel during Air-Drying

**Figure 4:**
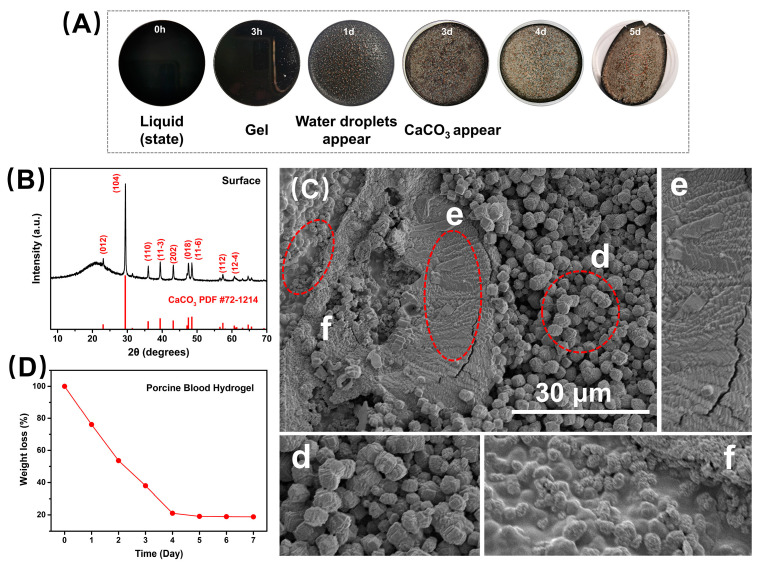
Illustrations of the formation of calcite on the surface of the porcine blood hydrogel. (**A**) Photos of the porcine blood hydrogel after standing for different times. (**B**) XRD pattern of the surface substance of the dried porcine blood hydrogel. (**C**) Surface morphology (×1.5K) (d, e, f corresponding position in (**C**)) and (**D**) weightlessness curve of the porcine blood hydrogel after standing for 5 days.

Figure 4A illustrates the changes in the surface morphology of the porcine blood hydrogel over time in plain air, where the porcine blood hydrogel could dehydrate to dry, which can be seen from its weightlessness curve with time exposed to the air (Figure 4D). The weightlessness was significant before 4 days and then extremely slow, indicating that the porcine blood hydrogel mainly lost free water before 4 days, and then subsequently lost the adsorbed water. The above dehydration characteristics may be related to the following factors: as the porcine blood hydrogel lost water, the hydrogel became hydrophobic, so a large amount of adsorbed water in the hydrogel was converted into free water, making the dehydration speed faster. In Figure 4A, we can see that there were dense water drops on the surface of the hydrogel air-dried for one day, proving the above conjecture. This is also consistent with the conclusion drawn from the interpretation of the contact angle experimental results mentioned above. When the blood gel was air-dried for 3 days (Figure 4A), white products appeared on the surface of the hydrogel, and subsequently, more and more white products were produced, possibly indicating that the lime water in the hydrogel was carbonated to form calcium carbonates. Of course, the carbonation of lime water in the porcine blood hydrogel’s surface could release water, causing the reverse effect of water loss. However, this effect cannot change the conclusion that the porcine blood gel becomes hydrophobic during drying.

To prove that the white surface product was the carbonated compound calcium hydroxide, it was examined using XRD. The corresponding results are shown in Figure 4B. Compared with the standard reference card, the characteristic peaks of the products formed on the surface were attributed to calcite. Obviously, the formation of surface calcite was related to the calcium hydroxide exposed to the surface of the porcine blood hydrogel to react with carbon dioxide in the air.

**Figure 5:**
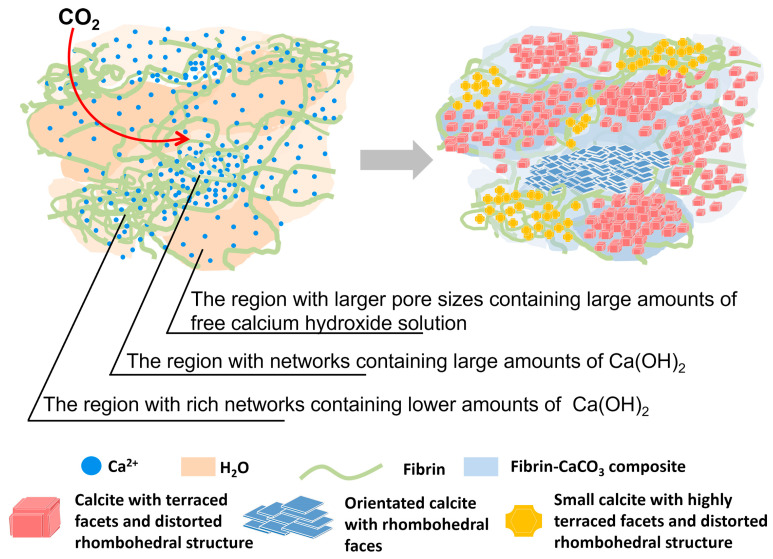
Diagram illustrating the different morphologies of calcite formed in different regions of the surface of the porcine blood hydrogel.

Figure 4C shows the SEM images of the surface morphology of the dried blood hydrogel after standing exposed to the air for 5 days. As a whole, due to differences in both the porcine blood hydrogel and the Ca(OH)_2_, three distinct crystal morphologies were formed. These morphological features were similar to that of calcite reported in the literature [43,44,45,46]. It has been found that the morphology of CaCO_3_ precipitates formed in hydrogels is dependent on the diffusion reaction [47]. In the porcine blood hydrogel, due to the uneven distribution of lime water and the difference in the diffusion of both Ca^2+^ ions and CO_2_, these formed calcite crystals with different morphologies that could be understood. In the porcine blood hydrogel, the region with larger pores possesses a more free calcium hydroxide solution, which makes many crystallized nucleuses easy to form. Consequently, small calcite crystallites with highly terraced facets and distorted rhombohedral morphologies appeared on the surface (Figure 4C(d)). For the region with networks of porcine blood hydrogel containing large amounts of Ca(OH)_2_, some carboxyl terminal groups of proteins adsorbed onto the (104) surface of the formed calcite nanocrystallites during the carbonation of Ca(OH)_2_. This suppressed any further growth of the individual crystallites, most likely along the (104) plane direction, resulting in the formation of calcite aggregates with layered ladder-like structures (Figure 4C(e)) [47]. This morphology of calcite crystals is similar to that reported in the literature [24,38,48]. For the region with rich networks of porcine blood hydrogel containing lower amounts of Ca(OH)_2_, the carbonation of limited Ca(OH)_2_ was more restricted by hydrogel networks, leading to the formation of small, grainy calcite crystals with round features (Figure 4C(f)), in agreement with the finding of previous studies [49]. Based on the interpretations mentioned above, the differences in the morphology of calcites formed in different regions of the surface of porcine blood hydrogels are shown in Figure 5.

### 2.3. Internal Carbonation in Porcine Blood Hydrogel during Air-Drying

**Figure 6:**
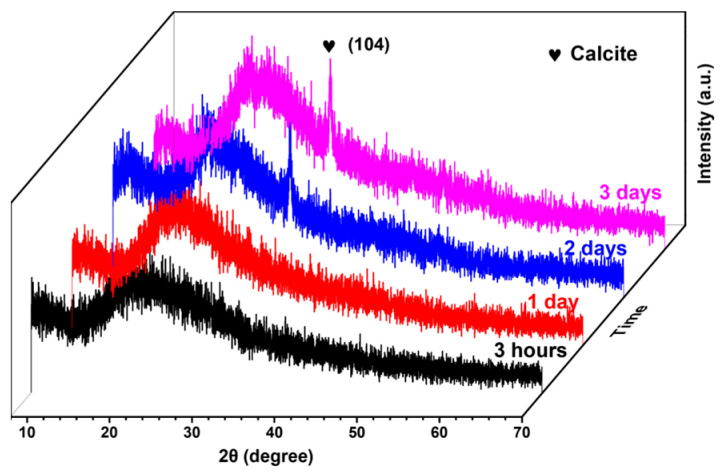
Variation in the XRD patterns of the porcine blood hydrogel after standing exposed to the air for different times.

Calcium carbonate primarily includes vaterite, aragonite, calcite, and amorphous calcium carbonate (ACC) [50,51,52,53,54]. ACC, characterized by its atomic arrangement lacking long-range periodic order, does not exhibit distinct diffraction peaks under X-rays due to the absence of a regular crystalline structure [19,55]. The XRD patterns of the porcine blood hydrogel samples after standing exposed to the air for 3 h, 1 day, 2 days, and 3 days are presented in Figure 6. For the samples that stood for less than 2 days, only one broad diffraction peak attributed to the amorphous protein structure appeared at a 2θ value of approximately 20.5°. The absence of characteristic diffraction peaks in the XRD patterns related to crystallized CaCO_3_ suggests that the calcium ions were free, bonded with ACC, or crosslinked with protein chains. However, for the samples that stood for more than 2 days, the characteristic diffraction peak emerged at 29.4°, corresponding to the (104) plane of calcite, indicating that the formation of calcite is likely to be closely related to the diffusion of both carbon dioxide and calcium ions depressed by the presence of the protein networks [18,56,57].

According to the difference in the results mentioned above regarding the carbonation that occurred on the surface and inside of the porcine blood hydrogel, it is not difficult to understand the roles of mass transfer and microenvironments during the carbonation process. Generally, the internal protein’s three-dimensional network structure in the sample restricts the nucleation, growth, and crystalline structure of calcium carbonate. Initially, the protein network structure induces the formation of ACC in areas of high calcium ion concentration, and then the metastable ACC is prone to dehydration, transforming into vaterite, which further converts into aragonite or calcite [55]. Additionally, this coordination between some carboxyl terminal groups (glutamic and aspartic acids) of proteins and Ca^2+^ not only forms specific crystal faces with calcium carbonate but may also be distributed within the calcium carbonate crystals [40,43,58]. The absence of distinct crystalline forms in the 3 h and 1 day samples suggests that ACC did not transform into vaterite, aragonite, or calcite. It is very likely that ACC forms in the porcine blood hydrogel due to the proteins having the ability to stabilize ACC [19,45,59].

**Figure 7:**
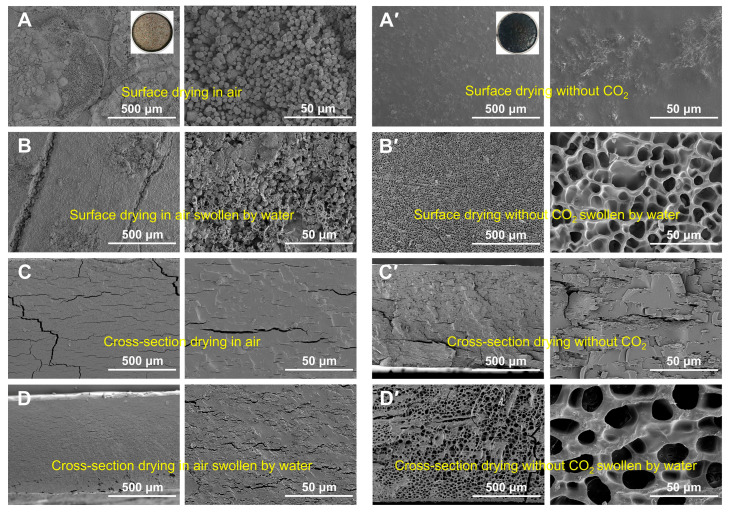
SEM images (×100, ×1K) of the porcine blood hydrogels air-dried (**left** column) in a CO_2_-free atmosphere (**right** column). The surface morphology of the sample before (**A**,**A’**) and after (**B**,**B’**) saturation with water. The cross-sectional morphology of the sample before (**C**,**C’**) and after (**D**,**D’**) saturation with water.

The surface and cross-sectional morphology of freeze-dried porcine blood hydrogel after being swollen by water are shown in Figure 7 (porcine blood hydrogels were air-dried in a CO_2_-free atmosphere). For the air-dried samples, the morphologies of their surfaces and the cross-sections changed slightly before and after soak with water (Figure 7A–D). Specifically, the calcium carbonate crystals still remained on the surface after water saturation, and the slight change in the morphology was mainly caused by the small amount of dissolved proteins. For the cross-sections of the corresponding sample before (Figure 7C) and after (Figure 7D) water saturation, we found that water saturation resulted in the formation of a few pores due to the hydrogel networks being partially swollen by water. Generally, a hydrogel is reversible in terms of water absorption. The dried hydrogel could be swollen by water, and the freeze-dried swollen hydrogel presented a porous structure. Clearly, the prepared porcine hydrogel was not a typical reversible hydrogel. We speculate that this irreversibility is related to the formation of calcium carbonate in the porcine blood hydrogel. Unavoidably, for the porcine blood hydrogel, Ca^2+^ in the interior of the alkaline hydrogel could react with diffused CO_2_ during air-drying. As a result, the formed calcium carbonate could inhibit the swelling of the hydrogel in water due to the interaction between calcium carbonate and proteins in the hydrogel.

The above conclusions were confirmed by the corresponding results of the dried hydrogels in the CO_2_-free atmosphere. For the dried samples in the CO_2_-free atmosphere, the morphologies of their surfaces and the cross-sections before and after water saturation are shown in Figure 7A’–D’. The results obviously indicate that the hydrogels dried in the CO_2_-free atmosphere possessed reversibility in terms of swelling. Combining the results here with those mentioned related to the air-dried porcine blood hydrogel, the following conclusion could be drawn that the non-reversibility of the water swelling of the air-dried porcine blood hydrogel can be attributed to the formation of CaCO_3_ in the porcine blood hydrogel.

**Figure 8:**
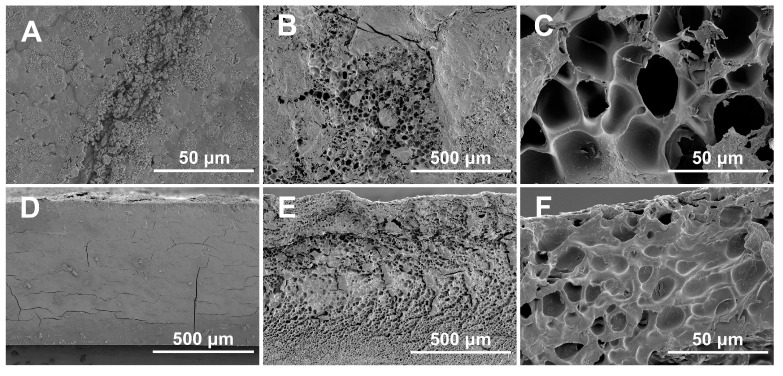
Surface (**A**–**C**) and cross-sectional (**D**–**F**) SEM images (×100, ×1K) of the air-dried porcine blood hydrogel before (**A**,**D**) and after (**B**,**C**,**E**,**F**) acid etching. The air-dried porcine blood hydrogel soaked with hydrochloric acid for 4 h.

To further verify the formation of calcium carbonate within the air-dried porcine blood hydrogel, the hydrogel was etched with a strong acid solution. The corresponding results are shown in Figure 8. It can be seen that the surface morphology of the blood hydrogel before (Figure 8A) and after (Figure 8B,C) acid etching exhibited a porous structure due to the granular calcium carbonate being dissolved by the acid. Similarly, the morphologies of the cross-sections of the blood hydrogel before (Figure 8D) and after (Figure 8E,F) acid etching also indicated a porous structure. The aforementioned results in Figure 7B,D show that the water-swelling ability of the air-dried porcine blood hydrogel is very limited. Therefore, the porous structure of the air-dried porcine blood hydrogel saturated with acid should be related to the calcium carbonate dissolved by acid.

**Figure 9:**
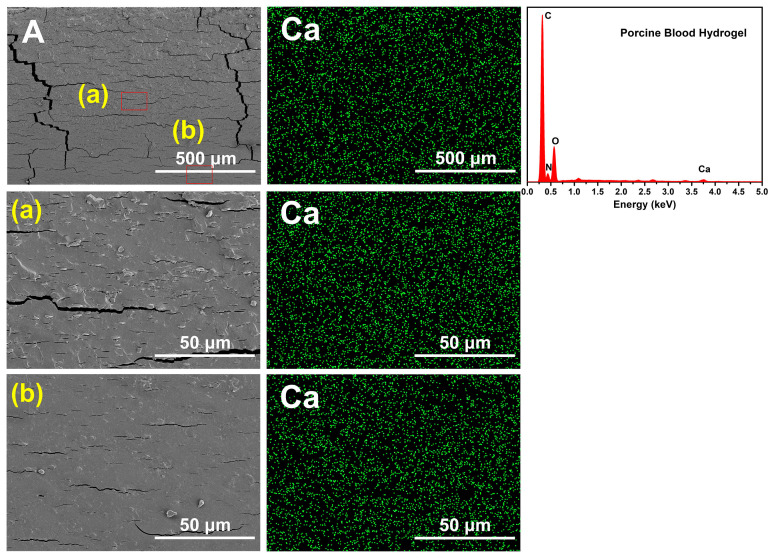
SEM images (×100, ×1K) and EDS spectrum as well as the corresponding Ca element mapping of the air-dried porcine blood hydrogel. a, b for the corresponding amplification in A.

It can be seen from Figure 8E that the porcine blood hydrogel after saturation with acid had a widely distributed dense pore structure, indicating that the amorphous calcium carbonate was more dominant in the porcine blood hydrogel than crystalline calcium carbonate. SEM images and EDS spectrum as well as the corresponding Ca element mapping image of the air-dried porcine blood hydrogel are shown in Figure 9. The distribution of elemental calcium was remarkably uniform, indicating that calcium-containing substances had a relatively uniform distribution in the porcine blood hydrogel. By comparing the widely distributed dense pore structure of the acid-saturated porcine blood hydrogel shown in Figure 8E and the distribution of elemental calcium shown in Figure 9, it can be concluded that there was widely distributed amorphous calcium carbonate in the porcine blood hydrogel.

### 2.4. Distribution Features of Carbonation in the Porcine Blood Hydrogel

**Figure 10:**
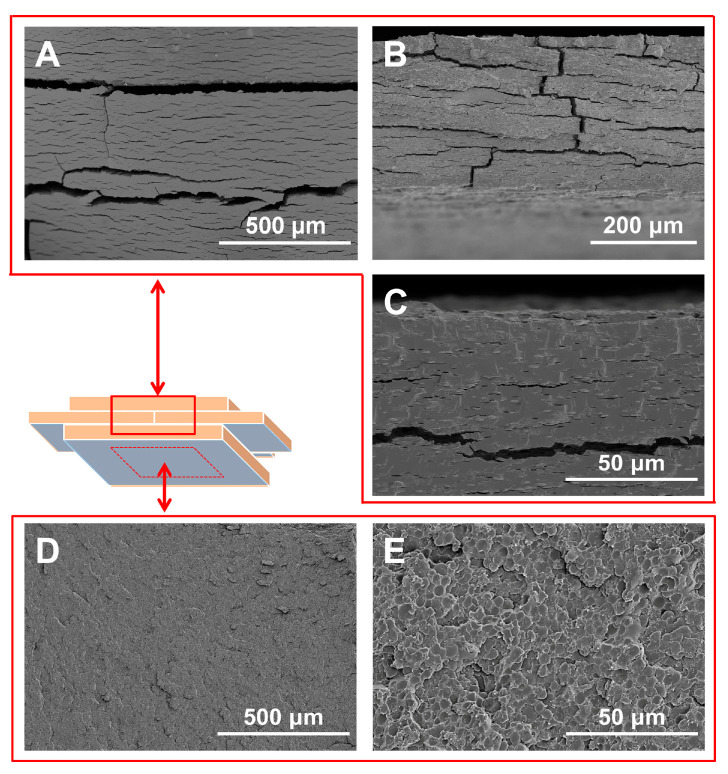
Cross-sectional SEM images (×100, ×200, ×1K) of the air-dried porcine blood hydrogel after standing in a humid atmosphere (**A**–**C**). (**B**,**C**) illustrate a local enlargement of (**A**,**B**), respectively; (**D**) illustrates the peeled lamellar surface; and (**E**) illustrates the local enlargement of (**D**).

Interesting lamellar morphological characteristics were accidentally found by observing the cross-sectional SEM image of the air-dried porcine blood hydrogel (Figure 10). It can be seen from Figure 10A that the cross-section of the air-dried porcine blood hydrogel after standing in a humid atmosphere showed a more obvious lamellar morphology. Compared with the air-dried porcine blood hydrogel shown in Figure 9, after placing the porcine blood hydrogel in a humid atmosphere for a certain period of time, this layered structure became more pronounced (Figure 10A–C). Additionally, these layers could be peeled, and the peeled lamellar surface exhibited a scale-like morphology, as shown in Figure 10D,E. According to the physical and chemical characteristics of the porcine blood hydrogel and the reaction process of Ca(OH)_2_ with diffused carbon dioxide in the porcine blood hydrogel, the formed lamellar structure is probably related to the formation process of calcium carbonate controlled by diffusion [60].

**Figure 11:**
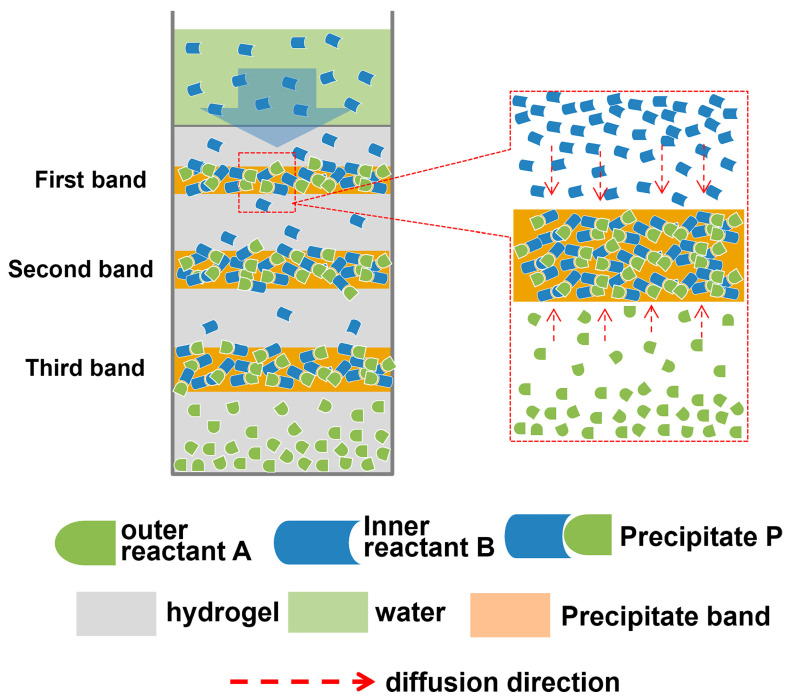
Diagram illustrating the formation principle of Liesegang patterns.

In fact, this finding is similar to those reported in the literature. Periodic precipitation formed in hydrogels is related to so-called Liesegang patterns (LPs) [61]. The formation of patterns is a transient non-equilibrium process driven mainly by reaction diffusion. In the typical formation of an LP in a hydrogel medium, initially, one of the reagents is homogeneously distributed in a hydrogel (the inner electrolyte, A), and after the gelation process, the solution of the other reagents (the outer electrolyte, B) is placed on top of the hydrogel column. When A meets B, due to the precipitation reaction (A(aq) + B(aq) → P (s)), an insoluble precipitate (P) forms. Under some experimental conditions, non-continuous periodic precipitation bands are produced. This principle is shown in Figure 11.

According to the principle of precipitation reactions, precipitate P is therefore formed when the ionic product of A and B ([A] × [B]) exceeds the critical value (ionic product constant, Ksp). If the precipitation reaction occurs in the hydrogel, the diffusion of both reactant A and reactant B is in a confined space. When the ion product of reactant A diffuses into the gel and reactant B encountered in the hydrogel is greater than Ksp, the precipitation reaction product is formed. Once precipitates form, their growth occurs by consuming the surrounding A and B. This occurs because the energy required for the formation of new nuclei is much higher than that needed for the growth of precipitates. As a result, both reactants A and B, located near precipitates, diffuse to the precipices for precipitate growth. This process proceeds until the local ion concentration is much less than the critical concentration. Large amounts of A and B are consumed in the initial precipitation area for the formation of precipitate bands. Consequently, the area near the initial precipitation area becomes the region with no precipitates due to the ionic product ([A] × [B]) being lower than Ksp. However, an area far from the initial precipitation area becomes the region where the ionic product ([A] × [B]) is higher than Ksp. A precipitate again forms when the ionic product ([A] × [B]) exceeds Ksp. By repeating these processes, intermittent precipitation bands (alternating precipitation and depletion regions) appear as Liesegang bands [62,63].

**Figure 12:**
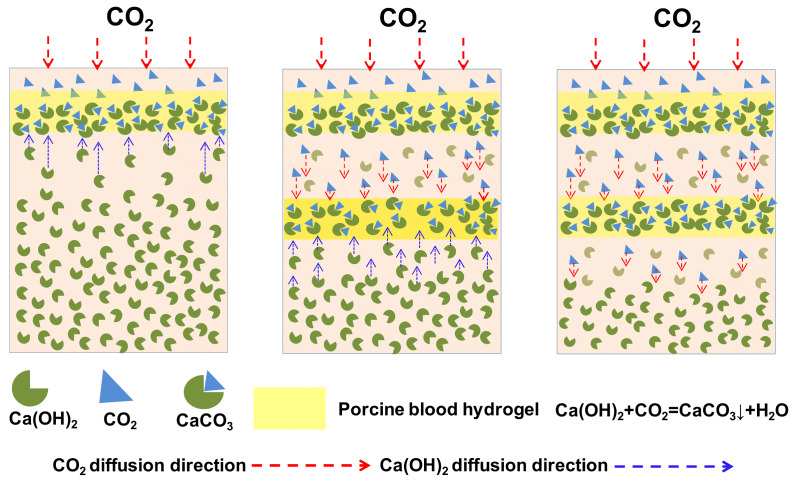
Diagram illustrating the formation principle of the lamellar patterns of calcium carbonate formed in the porcine blood hydrogel.

According to the aforementioned formation principle of Liesegang bands, it could be understandable that the cross-section of the porcine blood hydrogel displays a lamellar structure. As shown in Figure 11, during the air-drying of the porcine blood hydrogel, the diffused CO_2_ can react with Ca(OH)_2_ in the porcine blood hydrogel to form calcium carbonate when the ionic product ([(CO_3_)^2−^] × [Ca^2+^]) exceeds the ionic product constant of calcium carbonate (Ksp, 4.8 × 10^−9^). Once the calcium carbonate precipitates form, with their growth, the mobile calcium ions in the area near the precipitates constantly migrate to the precipitates to supply the calcium ions needed for the growth of the calcium carbonate precipitates. This process proceeds until the mobile calcium ions near the calcium carbonate precipitates are completely consumed. As a result, the band of calcium carbonate precipitates and the region lacking calcium carbonate precipitates form. By repeating these processes, intermittent precipitation bands appear as Liesegang bands with a lamellar structure. The formation principle of the lamellar patterns of calcium carbonate formed in the porcine blood hydrogel is shown in Figure 12.

It is precisely because the formed calcium carbonate exhibits a lamellar distribution in the porcine blood hydrogel that there must be an interface between the calcium carbonate-rich phase and the protein-rich phase. Due to the poor water swelling of the calcium carbonate-rich phase and the strong water swelling of the protein-rich phase, there is significant stress at the interface between the two phases in a humid atmosphere, which makes the two phases easy to delaminate. As predicted, the porcine blood hydrogel left to stand in a humid environment for a certain time appeared flaky, peeling after being freeze-dried (Figure 10A,B). Clearly, the peeled lamellar surface presented a flattened pore structure and some traces of brittle fracture along the edges of the pores (Figure 10D,E). The pore structure is related to the deposition of calcium carbonate on the pore walls of the porcine blood hydrogel, and the flattening of the pore structure can be attributed to the longitudinal shrinkage of the porcine blood hydrogel caused by drying dehydration. The longitudinal shrinkage can also be confirmed by the results shown in Figure 10C. The fact that the cross-section presents many small olive-shaped morphologies is the result of the longitudinal compression of the pore structure of the porcine blood hydrogel [64]. It is worth noting that the absence of a pore structure is related to the filling of those pores with calcium carbonate. As shown in Figure 10E, the brittle fracture surface with the pore structure indicates that the fracture occurs at the two-phase interface rich in calcium carbonate and proteins. By comparison, because the pores of the porcine blood hydrogel are filled with large amounts of calcium carbonate, the cross-section of the calcium carbonate-rich phase presents a smooth, brittle fracture feature with few pores (Figure 10C) [53].

## 3. Conclusions

In the ancient Chinese recipe for composite mortar used in the construction of ground layers for architectural paintings, the mixture of porcine blood and lime water is one of the constituent materials. In this case, the composite mortar is used to prepare a hydrophobic coating, and the mixture of porcine blood and lime water should have a special formula to meet the demands of the composite mortar. According to the traditional recipe, the interactions between porcine blood and lime water were systematically and deeply investigated. Based on our investigation, the following conclusions can be drawn:

(1) Porcine blood mixed with lime water at the ratio found in the recipe can form a hydrogel, and the formed hydrogel has a hydrophobic surface. It can be predicted that these features may be potentially beneficial not only for easily dispersing porcine blood in hydrophobic tung oil in the preparation of composite mortar but also for enhancing the affinity between porcine blood and hydrophobic tung oil.
(2) The lime water in porcine blood hydrogel can form calcium carbonate, nanocrystalline calcium carbonate, and amorphous calcium carbonate (ACC) in plain air. Calcite crystallites with highly terraced facets and distorted rhombohedral morphologies are formed when Ca^2+^ ions are present in a region with larger pores. In regions with networks of porcine blood hydrogel containing large amounts of Ca(OH)_2_, calcite aggregates with layered, ladder-like structures can form. Small, round calcite crystals are produced by Ca^2+^ ions in a region with a rich network of porcine blood hydrogel containing a lower amount of Ca(OH)_2_. ACC is primarily formed when Ca^2+^ ions are confined within hydrogel networks.
(3) Lime water in porcine blood hydrogel can become calcium carbonate via standing in plain air, and the formed carbonate can be used as a filler in the composite mortar to enhance its mechanical performance.
(4) The calcium carbonate formed in the porcine blood hydrogel exhibits a lamellar distribution. As a constituent material dispersed in the composite mortar, the layered distribution of calcium carbonate in the porcine blood hydrogel may be beneficial to reduce the internal stress of the composite mortar material.

These findings are particularly important for a profound understanding of the role of the porcine blood hydrogel in composite mortar used in the construction of ground layers for architectural painting and, therefore, its properties for future applications.

## 4. Materials and Methods

### 4.1. Materials

Fresh porcine blood was purchased from a slaughterhouse in the suburbs of Xi’an and was used directly, without treatment. Calcium oxide (CaO, AR), calcium chloride (CaCl_2_, AR), sodium hydroxide (NaOH, AR), and hydrochloric acid (HCl, AR) were acquired from Sinopharm Reagent Co., Ltd. (Beijing, China). The water used in the experiment was ultrapure water. These reagents were selected with reference to previous experimental results [65].

### 4.2. Preparation of Carbonized Porcine Blood Hydrogel

According to the recipe for using porcine blood as an additive material in the ground layer for traditional Chinese architectural paintings, the porcine blood was treated as follows: 25 g of calcium oxide was gradually added to 100 g of deionized water with continuous stirring to prepare the lime water; then, 4 g of suspended lime water was slowly added dropwise to 100 g of blood, with stirring. After stirring for 5 min, the mixture was left to stand for 3 h. As a result, a brownish-black porcine blood hydrogel formed. The fresh hydrogel was treated and freeze-dried to observe its network structure using SEM. Unless requirements specify otherwise, the porcine blood hydrogel should generally be kept exposed to the air for more than 5 days to dry.

### 4.3. Preparation of Samples Used in the Investigation

To test the carbonation and distribution of lime water in the porcine blood hydrogels, the following samples were prepared. The air-dried porcine blood hydrogels were etched in acid for 4 h. The porcine blood hydrogels etched by the acid were washed with ultrapure water and then freeze-dried. For purposes of comparison, samples soaked with water instead of acid were prepared. The surfaces and cross-sectional morphologies of the prepared samples mentioned above were observed using a scanning electron microscope. In these experiments, 0.5% diluted hydrochloric acid was used.

To investigate the factors related to the formation of the porcine blood hydrogel in the presence of lime water, some mixtures with different compositions were prepared, as shown in Table 1.

Specifically, to explore the carbonation of lime water in the porcine blood hydrogel, the two samples were prepared, including the two fresh porcine blood hydrogels, and were respectively placed in air and carbon-dioxide-free atmosphere. The detailed processes were as follows. For the first sample, porcine blood hydrogels were air-dried. For the second sample, quicklime was placed in a desiccator to dry in an atmosphere absent of carbon dioxide, and then the freshly formed porcine blood hydrogel was placed in the abovementioned desiccator for 5 days to dry. The difference in the cross-sectional morphologies of the above-formed samples were respectively observed using SEM.

### 4.4. Analytical Method and Instrumentation

The hydrophilic and hydrophobic properties of the relevant samples were detected using an OCA-20 optical contact angle meter (Shanghai Zhongchen Digital Technology Apparatus Co., Ltd. Shanghai, China). Each sample was detected three times, and images of water droplets at 10 s, 60 s, and 120 s were recorded. The samples for measuring the contact angle are powdery. The method for preparing the sample used to determine the contact angle is as follows: completely cover the surface of the adhesive tape with a layer of powder sample to be tested to make a contact angle measurement sample. Static contact angles of all samples were obtained using the sessile drop method, and contact angles were evaluated at room temperature with a model liquid (water) for all samples.

A high-resolution X-ray diffractometer (XRD, JDX-3532, JEOL, Tokyo, Japan) was employed to analyze both the surface and internal crystalline structures of the samples. The conditions were as follows: Using a Cu-target Kα X-ray source, the working current and the voltage were 30 mA and 10 kV, respectively. The step angle was set at 10°/min, with an angular range of 2θ from 8 to 70°. Jade 6.0 software was used for data analysis.

The morphologies of the samples were observed using an SU-3500 scanning electron microscope (SEM, Hitachi, Tokyo, Japan) at a signal of secondary electrons and voltages of 5 kV or 10 kV. All air-dried samples were mechanically broken into cross-sections for observation; freeze-dried samples were not required. The samples were coated with gold for 3 min before SEM analysis. Energy-dispersive spectroscopy (EDS) and elemental mapping were performed with the SEM to record the distribution of elemental calcium.

The pH of various solutions involved in Table 1 was measured using a Sartorius PB-10 professional pH meter (Sartorius AG, Gottingen, Germany). For sample A, the pH of porcine blood was determined; for samples B to D, the pH of the corresponding mixture after preparation was immediately determined.

## Figures and Tables

**Table 1:** The compositions of the samples for investigation of the factors related to the formation of porcine blood hydrogel.

Samples	Blood (g)	1M CaCl_2_ (mL)	Saturated NaOH (mL)	Lime Water (g)	Form	pH
A	100	6	/	/	sol	7.56 ± 0.04
B	100	/	2	/	hydrogel	11.86 ± 0.63
C	100	6	3	/	hydrogel	12.95 ± 0.03
D	100	/	/	5	hydrogel	12.73 ± 0.07

## Data Availability

The data that support the findings of this study are available from the corresponding author upon reasonable request.
